# Caregiving Through the Pandemic: Exploring the Impacts of COVID-19 on Caregivers and Their Caregiving Experiences

**DOI:** 10.1093/geroni/igab046.700

**Published:** 2021-12-17

**Authors:** Alexa Balmuth

**Affiliations:** MIT AgeLab, Cambridge, Massachusetts, United States

## Abstract

In the midst of the COVID-19 pandemic, many caregivers have been tasked with a unique role; not only to keep themselves safe and healthy, but also to protect and find new ways to aid their care recipients, many of whom are older adults at relatively high risk for severe complications from COVID-19. These challenging circumstances have driven caregivers to quickly adapt as they continue to manage their personal lives and caregiving responsibilities. Utilizing three waves of survey data from the MIT AgeLab Caregiver Panel, this presentation will examine the attitudes, experiences and worries of family caregivers at several time points along the course of the COVID-19 pandemic, as well as caregivers’ preparations and coping behaviors along the way. Differences between caregiving situations will also be discussed.

